# Personalized Medicine for Chronic Diseases Through the Integration of Health Determinants Control in Patients: A Systematic Review

**DOI:** 10.3390/jpm15100462

**Published:** 2025-09-30

**Authors:** Matthieu Bremond, Marie-Charlotte Raigneau, Joévin Burnel, Maxime Pautrat

**Affiliations:** 1 Department of Education and Training Sciences, University of Tours, 37044 Tours, France; 2 EA7505 Education Ethique Santé, University of Tours, 37044 Tours, France; maxime.pautrat@univ-tours.fr; 3 Institut d’ingénierie de la Santé, Université Picardie Jules Verne, 80000 Amiens, France; sos.mcraigneau@gmail.com; 4 Unité de Recherche en Sciences de la Réadaptation, Faculté des Sciences de la Motricité, Université Libre de Bruxelles, 1050 Brussels, Belgium; joevin.burnel@ulb.be; 5 Faculty of Medicine, University of Tours, 37044 Tours, France

**Keywords:** chronic disease, Health Locus of Control, self-efficacy, health determinant, quality of life

## Abstract

**Background**: Chronic disease significantly contributes to global healthcare demands and costs. Despite these chronic illnesses, good health is achievable through public health strategies that enhance control over health determinants. This systematic review investigates how control over health determinants affects the health status of individuals with chronic diseases. **Objective**: To assess the impact of limited control over health determinants on health status in people with chronic diseases and identify potential clinical applications. **Methods**: A systematic review was conducted following PRISMA 2020 and COSMOS-E guidelines. Searches across five databases (PubMed, Google Scholar, ScienceDirect, CINAHL, PsycARTICLES) between February and April 2023 identified cohort studies published in the last 10 years. Studies involving individuals aged 16 years and older with at least one chronic disease were included. The Newcastle–Ottawa scale was used to assess study quality. **Results**: Four cohort studies (*n* = 576) were included, involving participants with chronic diseases such as COPD, diabetes, and Parkinson’s disease. The methodological quality averaged 6/9. Significant correlations were observed between control over four health determinant domains—social, behavioral, biological, and healthcare system—and declining health outcomes. Common biases included detection and comparability bias. **Discussion**: The studies had acceptable methodological quality and low external bias risks. However, the meta-analysis was compromised due to the heterogeneity observed in the exposure variables of the included articles. The review emphasizes the importance of integrating control over health determinants into patient care, with healthcare professionals positioned to enhance patient control and improve outcomes. **Conclusions**: Lack of control over health determinants, particularly in social and behavioral domains, correlates with poorer health outcomes in patients with chronic conditions. Assessing and improve healthcare control could identify high-risk patients and improve their quality of life.

## 1. Introduction

**Figure 1:**
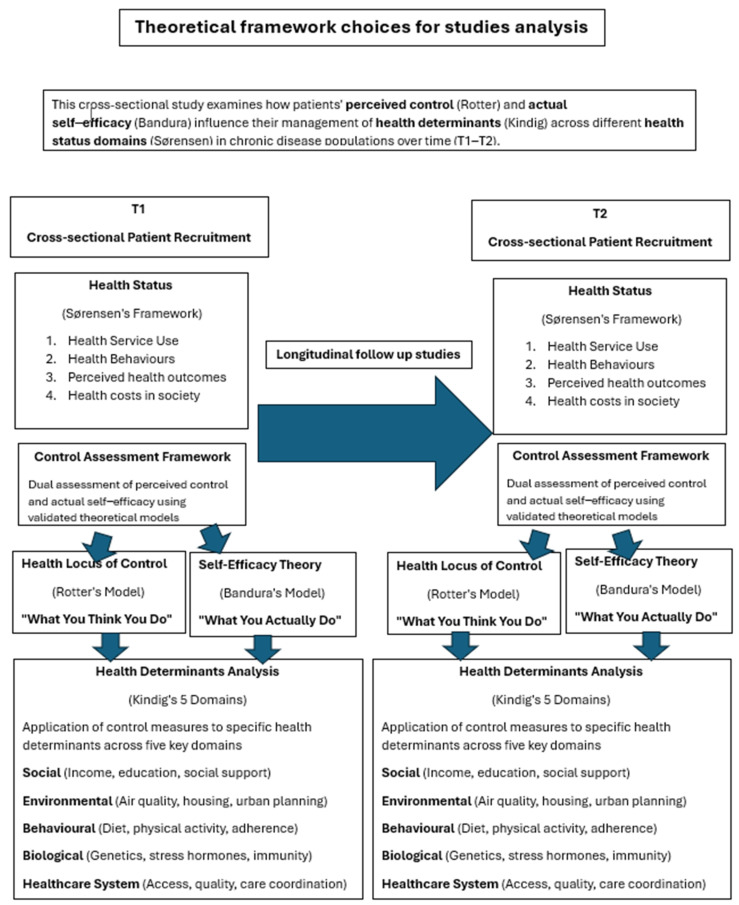
Theoretical framework choices for study analysis.

Chronic diseases account for approximately 41 million deaths annually, making up 74% of global mortality [1]. In France, two-thirds of deaths in 2018 were attributed to chronic conditions [2]. Chronic disease is characterized by a long-term pathological state associated with functional limitations and care dependency that affects daily life, yet individuals still retain some level of health potential [3]. This concept is known as “health-within-illness” [4,5] and has been described in several models. Kindig described five health determinant domains that influence health status, social (e.g., income, education, social support), environmental (e.g., air quality, urban planning), behavioral (e.g., diet, physical activity), biological (e.g., stress hormones), and healthcare system-related determinants [6]. Health determinant control has been described as two distinct dimensions [7,8,9]. Firstly, Bandura described a Self-Efficacy Theory, concerning the individual’s capacity to take concrete actions to control their health outcomes (“what you do”) [10]. Secondly, Rotter’s Health Locus of Control framework concerning the individual’s psychological sense of control over their health (“what you think you do”), which shapes their beliefs about their ability to influence their health trajectory and their motivation to engage in health-promoting behaviors [11]. Furthermore, Sørensen provided a more complex, four-domain health outcome framework to measure health status [12]. These models together measure the effect of key health determinant control on health status among patients living with chronic illnesses (Figure 1). Having an understanding of this less-explored concept is essential to understand and improve health outcomes in this population. The primary aim of this systematic review is to assess the impact of health determinant control on the health status of patients with chronic diseases.

## 2. Materials and Methods

This systematic review was conducted according to COSMOS-E guidelines for systematic reviews and meta-analyses of observational etiological studies [13] and reported according to the PRISMA statement [14]. A systematic article search was conducted in PubMed, Google Scholar, ScienceDirect, CINAHL Complete, and PsycARTICLES from the last decade (January 2013 to June 2025). Google Scholar was included as a complementary database alongside more specialized scientific databases specifically to capture gray literature and ensure comprehensive coverage of the available evidence. Registrar: The review is registered on PROSPERO (CRD42023378475).

Keywords were chosen from Health terminology /Ontology Portal (HeTOP) and MeSH terms: “chronic disease”, “Empowerment”, Self-Management”, “self-care”, “health status”. The search equation and the table are available in the appendix. No language restriction was applied.

Longitudinal cohort studies involving individuals aged 16 years and older with at least one chronic disease, in which participants either declared or actively controlled their health and published between 2013 and 2023, were included [6,11,15]. In accordance with current systematic review methodology and PRISMA guidelines, methodological quality was not used as an exclusion criterion. This approach avoids the risk of excluding potentially relevant studies whose data may contribute meaningfully to the synthesis, despite varying quality levels. Quality assessment was performed post-selection using the Newcastle–Ottawa scale to transparently report the quality of included evidence while avoiding the potential bias of excluding lower-quality studies that might still contribute valuable data to the field.

Studies that involved a substantial proportion of participants with multiple chronic conditions were excluded unless a subgroup analysis was accessible. These studies were excluded as health status deteriorates differently among chronic diseases and combinations of chronic diseases, preventing confounding factors that could affect result interpretation.

Four outcome criteria stood out from the eight-domain Sørensen framework—healthcare services, behaviors, costs, and perceived health outcomes [12]—and these refer to four dependent variables that describe health status. These variables referred to as health indicators are those statistically significant effect measures (odds ratio) that assess an individual’s health status independent of disease presence [3]. Any health indicator that did not relate to a Sorensen category were disregarded.

Those studies that examine social networks and measure the frequency of social interaction as a health determinant were classified as “social” rather than “behavioral” according to Kindig’s framework. The associate health indicators were categorized as “social support” rather than “cognitive capacity” because they specifically measure the presence and frequency of supportive interactions, rather than a mental process or skill. Furthermore, Bandura control type was classified as “action” rather than “perception” since the frequency of interaction reflects a tangible behavior (an action the individual performs), rather than their subjective perception of control over their social environment.

This process ensures that variables were accurately mapped to the appropriate health determinants and control types, facilitating a clearer understanding of the relationship between exposure and health outcomes.

### 2.1. Data Collection and Analysis

Studies were collected during a blinded three-stage selection process during which titles, abstracts, and then full texts were screened for eligibility using Rayyan^®^ software (2016) 5:210. Duplicates were discarded. Two authors independently selected the studies a third party arbitrated if needed and the final selection was validated by consensus.

Data extraction and management: Articles were exported to Zotero 7.0.16 and uploaded to Rayyan for selection and data extraction. Author, publication year, country, study objective, design, population description, exposure description, outcome description and indicators, statistical results, clinical relevance, and funding source were extracted. Discrepancies were resolved through discussion. A critical review assessed external, statistical validity, and clinical relevance, and internal validity was assessed using The Newcastle–Ottawa Quality Assessment Form for Cohort Studies.

The unit of analysis for health determinant control or lack of control from included studies were events that indicate a deterioration in health status. Studies reported these health status events as odds ratios (OR) or log odds ratios (Beta coefficients). As the unit of analysis, particularly the independent variables, were clearly heterogeneous from the outset, a meta-analysis was not planned.

The heterogeneity between selected studies was assessed by mapping perceived or active control over four types of health determinants (biological, social, behavioral, and health system).

### 2.2. Summary of Conclusions and Assessment of the Certainty of the Evidence

All tables including statistically and clinically significant outcomes will be listed in a summary of results table, correlated with exposure and its clinical meaning.

### 2.3. Protocol Amendments

Participant selection was revised to include people aged over 16 years.

### 2.4. Summary Box

What is already known on this topic?

Chronic diseases significantly impact global health, but controlling health determinants can influence the health status of people with chronic diseases.

What is added by this report?

This systematic review shows that greater control over social, behavioral, and emotional health determinants is consistently associated with improved health outcomes and reduced healthcare use in people with chronic conditions.

What are the implications for public health practice?

Integrating assessment and support of patient control into chronic care strategies could enhance self-management, lower healthcare costs, and improve long-term outcomes.

## 3. Results

**Figure 2:**
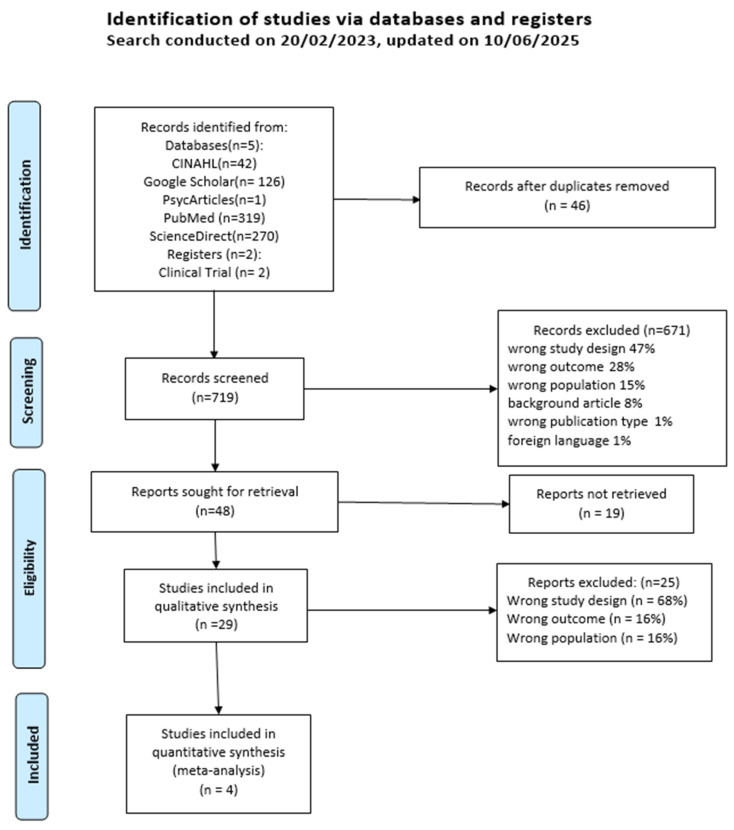
PRISMA Flow Chart.

This study describes four health determinants: healthcare service use, health behaviors, healthcare costs, and perceived health outcomes. Figure 2 illustrates the study flow chart. Overall, 576 articles were identified and four studies were included and analyzed.

Table 1 describes the terminology used in each identified study to describe health determinant variables alongside the associated health outcome variables [16,17,18,19]. The health determinants were classified according to health determinant descriptions. A notion of control for illness perception, personal resources, self-care confidence, and disease management was found in two of the four studies included.

Table 2 shows those health indicators that relate to the Kindig health determinants [6] and either perception (what the patient thinks) or action (what the patient does); Bandura [15] and Rotter [11] control type. Notably, two studies, Rijken et al. and Reeves et al., reported participants having perceived control over social networks, while the other three studies assessed a broad spectrum of health determinants with either perceived or active Bandura control. Some determinants were assessed with greater granularity than others (i.e., with a greater number of indicators) and a range of 0 to 6 independent variables was found for each control type. Also, not all health determinant types were reported in all studies or even represented with the same indicator or control type. Overall, a total of 47 independent variables were considered relevant, of which 26% were social determinants, 17% health system determinants, 47% behavioral determinants, and 11% biological determinants; 32% of control type were perceived and 68 were active.

Health outcome measures were identified from Sørensen et al.’s healthcare domains [12] and are listed in Table 3: one health service use, two health behaviors, three health costs, and four perceived health outcomes. No studies evaluated the remaining four domains, which concern health literacy.

Table 4 lists the correlation between health status outcomes and Kindig health determinant control. Biological and social determinants had significant impacts on health status outcomes. Notably, a lack of social network control correlates with frequent emergency room visits, avoidable hospitalizations, lower quality of life, increased primary care contact, higher health costs, and less beneficial health behaviors. One notable positive example was found in Rijken’s study, which measured the frequency that social networks were mobilized to support the patient’s quality of life.

Conversely, a lack of social network involvement (low social determinant control) significantly correlated with higher healthcare costs and less-frequent beneficial health behavior engagement [16]. This correlation suggests that patients who are less able to mobilize their social networks to assist with disease management experience worse health outcomes. This is measured by more frequent hospitalizations and lower quality of life.

Furthermore, a positive biological determinant, such as emotional control, was significantly associated with favorable health outcomes as indicated by increased general medicine use outside the office. Also, physical health significantly improved quality of life and reduced avoidable outpatient and emergency hospitalizations, whereas negative emotional control such as depression was significantly associated with frequent GP contact and reduced quality of life.

**Figure 3:**
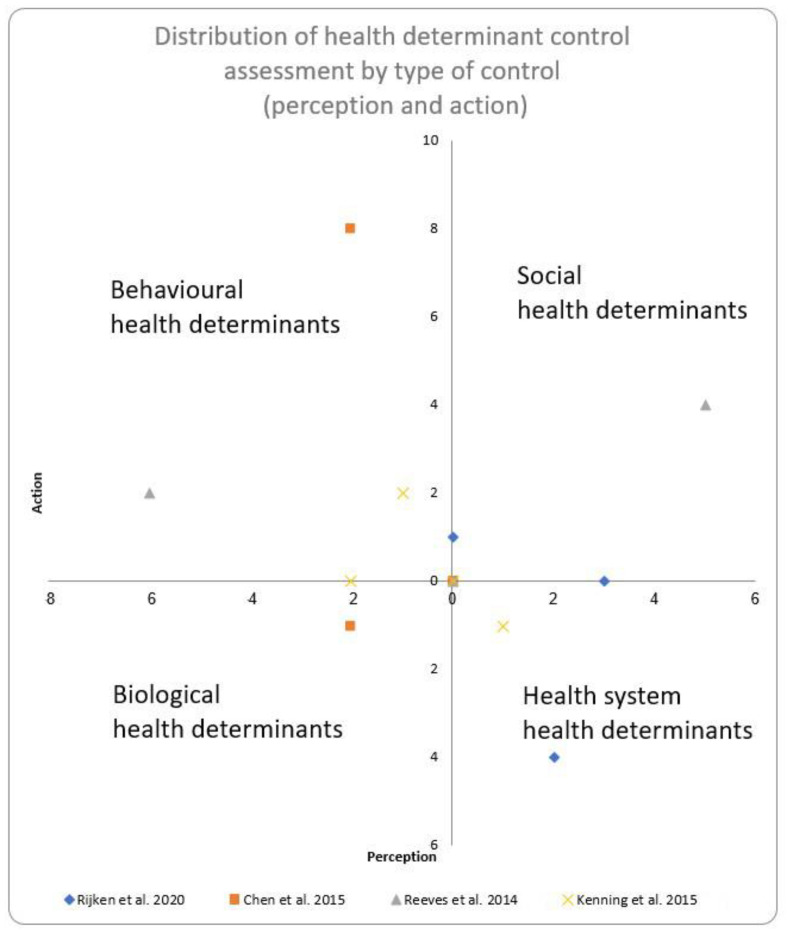
Representation of heterogeneity described in Table 2 based on studies retained in the systematic review [16,17,18,19].

Furthermore, the assessment of health determinant control among the studies was considerably heterogeneous. This heterogeneity is illustrated in Figure 3, in which each dot represents the type of control an individual may have over biological, social, behavioral, and health system health determinants associated with the participant-reported perceived or active control (Figure 3 described in Table 2). Each dot position in the quadrant is a function of the number of health indicators for which participants reported as having either perceived control on the x-axis or active control on the y-axis. Each color represents a study. Only the center point overlaps when the study does not describe the determinants. This heterogeneity between studies makes it difficult to collate the independent variables.

### Risk of Bias in Included Studies

Each included study was appraised using the Newcastle–Ottawa scale.

Table 5 describes the methodological quality of the studies included. The risk of bias was low in two studies (Rijken and Reeves) and high for Kenninga and Chen.

**Figure 4:**
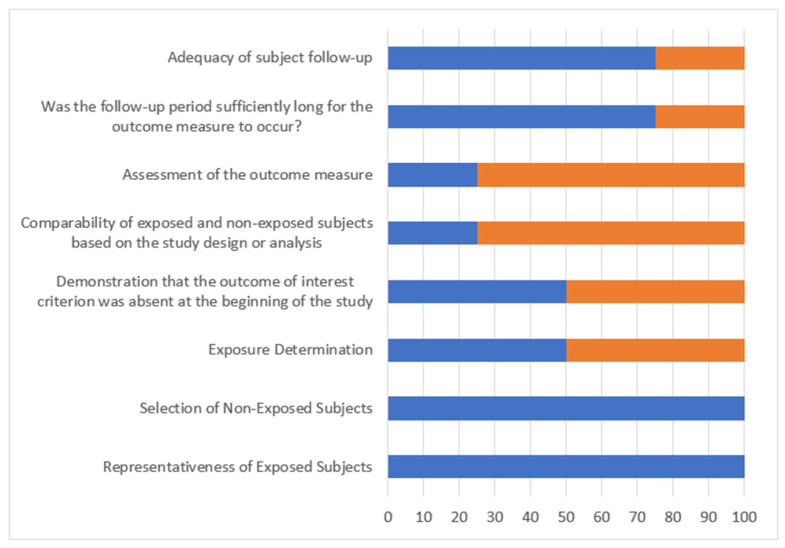
Newcastle–Ottawa risk of bias assessment graph (proportion of risk in orange color). The blue color represents the proportion of studies in which the risk of bias (named in the first column) was controlled/addressed in the studies included in the systematic review (and the orange was not controlled).

The assessment of the methodological quality of the articles is summarized in Figure 4. The methodological quality assessed using the Newcastle–Ottawa scale ranged from four to eight stars out of nine possible stars.

## 4. Discussion

The main finding of this study is the significant impact that control over key health determinants has on improving health outcomes in patients with chronic illnesses. In particular, we found that control over social determinants, such as social support [6], led to substantially improved perceived health outcomes and notably reduced healthcare costs [12]. This was achieved through action-based control [15], where patients actively engaged their social networks, leading to fewer emergency room visits and avoidable hospitalizations.

Additionally, control over behavioral determinants, like regular physical activity and dietary habits, directly contributed to enhanced health behaviors and improved quality of life [12]. Patients who took active steps to manage their health behavior not only reduced healthcare use but also reported higher levels of well-being.

Furthermore, the studies revealed that lack of control over emotional responses to illness, such as depression, was strongly correlated with increased primary care visits and worsened mental health outcomes. Alternatively, patients who maintained control over their emotional and cognitive responses showed better self-management of their disease, leading to lower health service dependency.

Overall, the results clearly demonstrate that empowering patients to take control of their social and behavioral determinants can significantly improve health outcomes, reduce costs, and enhance the quality of life, making these areas crucial targets for intervention in chronic disease management.

The limited number of eligible studies identified highlights a significant gap in the literature and demonstrates the need for more focused research in this area, which we discuss as a key limitation and direction for future research.

The quality of evidence was moderately high. The methodological quality assessed using the Newcastle–Ottawa scale ranged from four to eight stars out of nine. Studies conducted in Europe and Taiwan varied in healthcare system organization, influencing generalizability. Populations in English studies were socially disadvantaged, affecting extrapolation. Statistical validity issues include lack of alpha risk weighting and power calculation. Despite these limitations, the results remain clinically relevant and highlight hypotheses for future practice.

### 4.1. External Consistency

The review confirms known relationships between physical activity, diet, mental health, and social support in improved health outcomes. Addressing the lack of health control can help curb health deterioration spirals, emphasizing the importance of evaluating and supporting control strategies in managing chronic illness.

### 4.2. Clinical Relevance

Assessing control over health-determining factors highlights the importance of the “control” dimension in health status deterioration. Integrating this aspect into care pathways can maximize health benefits for chronically ill patients.

### 4.3. Strengths and Limitations

This study offers several strengths that enhance its contribution to patients understanding the impact of control over health determinants in patients with chronic diseases. First, the adherence to PRISMA and COSMOS-E guidelines ensures a rigorous and transparent systematic review process, which enhances the reliability and reproducibility of the findings. The inclusion of studies spanning a decade across multiple reputable databases adds to the comprehensiveness and breadth of the literature reviewed. A limitation of this article lies in the authors’ choice of an exploratory framework that focuses on only four of Sørensen’s eight health outcome domains, while applying the Kindig and Bandura models to classify and interpret health control. This selective approach may omit certain aspects of health status and control, potentially limiting the comprehensiveness of the findings in the context of chronic illness management. Furthermore, the methodological quality of the included studies, assessed using the Newcastle–Ottawa scale, indicates a satisfactory level of evidence, providing confidence in the overall conclusions drawn. However, the study also has notable limitations. The heterogeneity among the independent variables and the health determinants assessed across the included studies precluded a meta-analysis, limiting the ability to quantitatively synthesize the data. Additionally, the lack of alpha risk weighting and power calculations in the statistical analyses of the included studies may introduce biases and affect the robustness of the results. The focus on developed countries with specific healthcare systems may limit the generalizability of the findings to other settings. Moreover, the exclusion of environmental and genetic determinants, which are difficult to control at an individual level, could overlook important factors influencing health outcomes. Despite these limitations, the study provides valuable insights and highlights areas for future research to further explore the relationship between control over health determinants and health status in chronic disease management.

Under these conditions, a meta-analysis did not seem relevant due to the heterogeneity observed in the reported health determinant descriptors in the articles included. A narrative summary of the results was applied.

## 5. Conclusions

Lack of control is associated with poorer health. In this context, is it the presence of the chronic disease or the lack of control over its health determinants that is primarily responsible for the initial health decline? Improving individual control is central for healthcare systems, requiring assessment to establish relevant management for chronically ill people. The limited number of studies and heterogeneity of indicators call for further cohort studies on this subject.

## Figures and Tables

**Table 1:** Description of health determinants identified in the included studies.

				Health Status Notions Identified in the Articles													
How the Health Determinants Were Described by Authors of Each Article	Variables Describing Control on Health Determinants, Listed in the Articles	Adjustment Factors (Confounders)	Tick if Articles Identify Some Form of Control over the Variables in the Second Column	Frequent Contact with GP	General Medicine Use Outside of Practice	Unplanned Hospitalizations	Poor Health-Related Quality of Life	Emergency Consultations	Planned Hospitalizations	Avoidable Hospitalizations	Self-Management	Health Behaviors	Physical Health	Emotional Well-Being	Cost of Health Services	QUALYs Over The Last 12 Months	Self-Assessment of Health Status
**Illness Perception**	Illness Consequences		X	Rijken et al., 2020, [19] Dutch aged 15 and over with at least 2 chronic conditions *n* = 747													
	Timeline		X														
	Personal Control		X														
	Treatment Control		X														
	Identity		X														
	Worry		X														
	Illness Understanding		X														
	Emotional Response		X														
**Personal Resources**	Education Level	X															
	Financial Management		X														
	Health Provider Support																
	Having Sufficient Information		X														
	Active Health Management		X														
	Social Support		X														
	Critical Evaluation		X														
	Active Engagement with Health Care Providers		X														
	Navigating the Health Care System		X														
	Ability to Find Good Health Information		X														
	Reading and Understanding Health Information		X														
	Mastery		X														
	Anxiety and Depression		X														
**Confidence in Self-care**	Adherence		X					Chen et al., 2015 [18], Taiwanese aged 65 and over, 80% with at least one chronic condition *n* = 2531									
	Exercise		X														
	Diet		X														
	Ability to Express Health Conditions		X														
	Ability to Solicit		X														
	Ability to Control Illness		X														
	Ability to Control Emotions		X														
**Disease Management Behavior (Past Year)**	Weight Control		X														
	Smoking		X														
	Alcohol		X														
	Exercise		X														
	Follows a Diet		X														
	Adaptation to Daily Life		X														
	Age	X															
	Gender	X															
	Education	X															
	Ethnicity	X															
	Marital Status	X															
	Employment Status	X															
	Living Conditions	X															
	Living Area	X															
	Number of Chronic Diseases	X															
	Fall Experience	X															
	Disabilities in Daily Activities	X															
	Depressive Symptoms		X														
	Cognitive Disorders		X														
	Self-assessment of Health	X															
	Gender	X									Reeves et al., 2014 [16], Heart failure or diabetic patients recruited in Greater Manchester *n* = 248						
	Age	X															
	Main Condition	X															
	Number of Long-term Conditions	X															
	Living Area Precarity	X															
	Ethnicity	X															
	Socio-professional Class	X															
	Education	X															
	Income	X															
**Characteristics of Network Members**	Number of Children Nearby	X															
	Contact Frequency	X															
	Number of Co-inhabitants																
	Partner																
**Characteristics of the Social Network**	Network Density	X															
	Network Variety	X															
	Participation in Local Support Groups	X															
	Support Given to Others	X															
	Social Resources	X															
	Support Provided by the Network for Illness Management	X															
	Emotional Support Provided by the Network	X															
	Network Support in Daily Tasks	X															
**Network Change Measures**	Loss of One or More Important Network Members	X															
	Loss of Network Support	X															
	Gender															Kenning et al., 2015 [17], Patients with at least 2 conditions among COPD, coronary diseases, diabetes, osteoarthritis, depression; recruited in Greater Manchester *n* = 410	
	Ethnicity																
	Professional Activity																
	Age																
	Living Area Precarity																
	Number of Long-term Conditions																
	Anxiety and Depression	X															
	Health Status	X															
**Care Services Experiences**	Experience in Care Organization	X															
	Care Concerns	X															
**Illness Perception**	Illness Consequences	X															
	Personal Control	X															
	Treatment Control	X															
	Experience of Multimorbidity	X															

**Table 2:** Kindig health determinants with health indicators associated with Bandura control type.

Determinant of Health Category (Kindig)	How the Determinant was Measured	What the Patient Thinks He does (Perception) Versus What He Actually Does (Action) (Bandura and Rotter)	Study	Number of Health Determinant Descriptors Found
Social Determinants	Income	Perception	Rijken et al. [19]	1
Social Determinants	Social Support	Perception	Rijken et al. [19]	1
Social Determinants	Locus of Control	Perception	Rijken et al. [19]	1
Social Determinants	Social Support	Perception	Reeves et al. [16]	5
Social Determinants	Social Support	Action	Reeves et al. [16]	4
Health System Determinants	Access to Care	Perception	Rijken et al. [19]	1
Health System Determinants	Access to Care	Perception	Kening et al. [17]	1
Health System Determinants	Access to Care	Action	Kenning et al. [17]	1
Health System Determinants	Access to Care	Action	Rijken et al. [19]	3
Health System Determinants	Quality of Care	Perception	Rijken et al. [19]	1
Health System Determinants	Quality of Care	Action	Rijken et al. [19]	1
Behavioral Determinants	Diet	Perception	Chen et al. [18]	1
Behavioral Determinants	Diet	Action	Chen et al. [18]	1
Behavioral Determinants	Physical Exercise	Perception	Chen et al. [18]	1
Behavioral Determinants	Physical Exercise	Action	Chen et al. [18]	1
Behavioral Determinants	Consumption	Action	Chen et al. [18]	2
Behavioral Determinants	Illness	Perception	Reeves et al. [16]	6
Behavioral Determinants	Illness	Perception	Kenning et al. [17]	1
Behavioral Determinants	Illness	Action	Reeves et al. [16]	2
Behavioral Determinants	Illness	Action	Kenning et al. [17]	2
Behavioral Determinants	Illness	Action	Chen et al. [18]	4
Behavioral Determinants	Illness	Action	Rijken et al. [19]	1
Biological Determinants	Anxiety and Depression	Perception	Kenning et al. [17]	1
Biological Determinants	Anxiety and Depression	Perception	Chen et al. [18]	1
Biological Determinants	Anxiety and Depression	Action	Chen et al. [18]	1
Biological Determinants	Cognitive Capacity	Perception	Chen et al. [18]	1
Biological Determinants	Health Status	Perception	Kenning et al. [17]	1

**Table 3:** Health status outcome according to the four Sorensen domains.

Health Outcome Measures
**Health Services Utilization**
Frequent Contact with GP
General Medicine Use Outside the Office
Unplanned Hospitalizations
Emergency Consultations
Planned Hospitalizations
Avoidable Hospitalizations
Outpatient Consultations
**Health Behaviors**
Self-management
Health Behaviors
**Health Costs**
Cost of Health Services
**Health Outcomes**
Physical Health
Emotional Well-being
QUALYs over the Last 12 Months
Poor Health-Related Quality of Life

**Table 4:** Main results of the influence of control of health determinants on health status.

Influence of Lack of Control Over Health Determinants on Health Status (Only Significant Results are Reported)				
Healthcare Outcome		Number of Studies	Study	Correlation
Health System Use		3		
	Primary Care	1	**Rijken et al. 2020** [19]	
			General medicine use outside surgery	(B)(**+**) Emotional reaction to the illness *
			Frequent GP contact	(B)(**+**) Worry *
				(B)(**−**) Ability to master the illness *
	Specialised care	1	**Rijken et al. 2020** [19]	
			Specialised care	(B)(**−**) Personal control **
		1	**Chen et al. 2015** [18]	
			Number of avoidable hospitalisations	(OR)(**−**) Initiated physical activity (95% CI: 0.43–0.82) ***
				(OR)(**−**) Self-care confidence (95% CI: 0.94–0.99) *
				(OR)(**+**) ADL disability (95% CI: 1.10–2.56) *
			Number of emergency consultations	(OR)(**−**) Initiated physical activity (95% CI: 0.59–0.96) *
				(OR)(**+**) Symptoms of depression (95% CI: 1.02–1.72) *
			Number of outpatient consultations	(OR)(**−**) Initiated physical activity (95% CI: 0.55–0.93) *
**Healthcare service costs**		1		
		1	**Reeves et al. 2014** [16]	
			Cost of health care services	(B)(**−**) Help the patient’s network provides to manage illness(SE = 8.61) **
			QUALYs (over the last 12 months)	(B)(**−**) Social involvement (SE = 2.34) **
**health behaviours**		2		
	Self-management	1	**Reeves et al. 2014** [16]	(B)(**+**) Support given to others (SE = 0.029) *
				(B)(**+**) Help the patient’s network provides to manage illness (SE = 0.002) *
				(B)(**+**) Social involvement (SE = 0.021) *
	Health Behaviour	1	**Reeves et al. 2014** [16]	(B)(**+**) Help the patient’s network provides to manage illness (SE = 0.007) *
**Health outcomes**		2		
	Quality of Life	1	**Reeves et al. 2014** [16]	
				(B)(**+**)Social involvement (SE = 1.05) **
				(B)(**+**) Help the patient’s network provides to manage illness (SE = 0.11) **
			Physical health	(B)(**+**) Help provided to others (SE = 0.56) *
				(B)(**+**) Social involvement (SE=0.37) *
		1	**Rijken et al. 2020** [19]	
			Quality of Life	(B)(**−**) Depression **
				(B)(**+**) Perception illness consequences **
				(B)(**+**) Personal control *
				(B)(**+**) Identity facing illness **
				(B)(**+**) “I have many people around me” *

No significant results for the Kenning et al. Study. B, coefficient B = log(odd ratio). (−) Negative association. (+) Positive correlation. CI Confidence interval. * *p* < 0.05. ** *p* < 0.005. *** *p* < 0.0001.

**Table 5:** Risk of bias: each element of the risk of bias for each included study (in green color the risk of bias is controlled in the study, in red color the risk is not controlled).

	Selection Biais				Comparability Bias	Detection Bias		Attrition Bias
	Representativeness of Exposed Subjects	Selection of Non-Exposed Subjects	Exposure Determination	Demonstration That the Outcome of Interest Criterion was Absent at the Beginning of the Study	Comparability of Exposed and Non-Exposed Subjects Based on the Study Design or Analysis	Assessment of the Outcome Measure	Was the Follow-Up Period Sufficiently Long for the Outcome Measure to Occur?	Adequacy of Subject Follow-Up
Rijken (2020) [19]	X	X	X	X	X X	X	X	X
Chen (2015) [18]	X	X	X	X	X X	X	X	X
Kenninga (2015) [18]	X	X	X	X	XX	X	X	X
Reeves (2014) [16]	X	X	X	X	XX	X	X	X

## Data Availability

The raw data supporting the conclusions of this article will be made available by the authors on request.
